# Skull Shape Diversity in Pet Rabbits and the Applicability of Anatomical Reference Lines for Objective Interpretation of Dental Disease

**DOI:** 10.3390/vetsci7040182

**Published:** 2020-11-20

**Authors:** Christine Böhmer, Estella Böhmer

**Affiliations:** 1 UMR 7179 CNRS/MNHN, Département Adaptations du Vivant, Muséum National D’Histoire Naturelle, CP 55, 57 rue Cuvier, 75005 Paris, France; 2 Chirurgische und Gynäkologische Kleintierklinik, Tierärztliche Fakultät, Ludwig-Maximilians-Universität München, Veterinärstr 13, 80539 München, Germany; e.boehmer@gmail.com

**Keywords:** Lagomorpha, geometric morphometrics, teeth, masticatory apparatus, pathology, malocclusion, evolution

## Abstract

Acquired dental problems are among the most frequently encountered diseases in pet rabbits. However, early symptoms are often overlooked because the affected animals first appear completely asymptomatic. Alterations from anatomical reference lines according to Böhmer and Crossley applied to standard skull X-ray images, have been shown to be indicative of tooth health problems in pet rabbits. Despite its proven usefulness, there are exceptions in which the anatomical reference lines appear not to be suitable for application. We addressed this issue by quantifying the cranial morphology of a large data set of pet rabbit patients (N = 80). The results of the morphometric analyses revealed considerable diversity in skull shape among the typical pet rabbits, but variance in only a few parameters influences the applicability of the anatomical reference lines. The most substantial parameter is the palatal angle. Specimens in which the anatomical reference lines could not be applied, have a rather large angle between the skull base and the palatal bone. We recommend to measure the palatal angle before applying the anatomical reference lines for objective interpretation of dental disease. Pet rabbits with a palatal angle larger than 18.8° are not strictly suitable for the successful application of the anatomical reference lines.

## 1. Introduction

**Figure 1:**
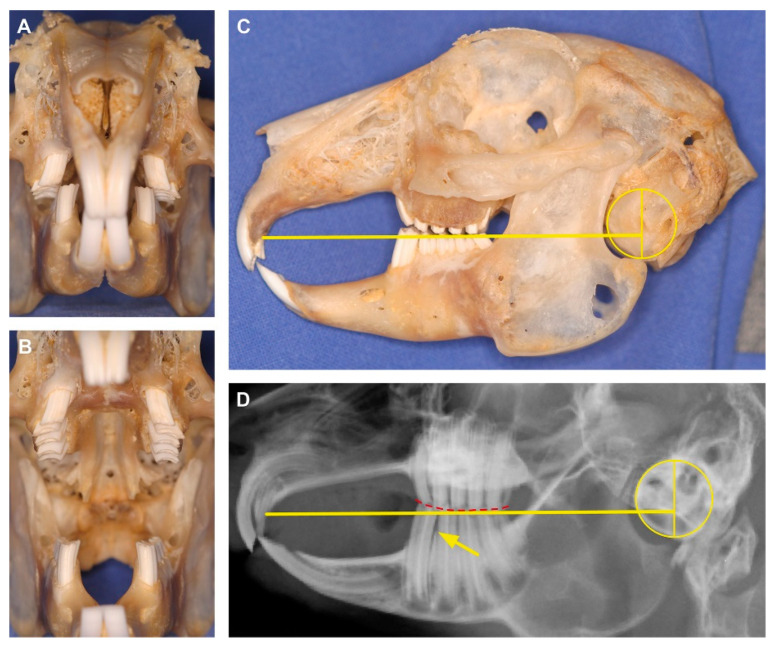
Example highlighting the importance to integrate effective, noninvasive techniques, such as radiography, in the clinical examination routine. (**A**) Rostrocaudal view of an apparently healthy dentition in a pet rabbit with mouth closed and (**B**) opened. (**C**) Lateral view of the skull with applied anatomical reference lines according to Böhmer and Crossley [11]. The yellow line begins at the rostral end of the hard palate immediately caudal to the second incisor and extends caudally to pass through the tympanic bulla at approximately one-third of its height. In healthy pet rabbits, it marks the physiological occlusal plane between maxillary and mandibular molars. In the present specimen, the occlusal line depicts a slight elongation of the clinical crown of the first lower cheek tooth (P3) which might be missed on clinical examination alone (see A and B). (**D**) The laterolateral X-ray is the gold standard to detect that this rabbit suffers from an early stage of malocclusion. The overlong mandibular P3 progressively bends rostrally causing a widening of the approximal space to the adjacent P4 (arrow). Due to the abnormally short clinical crown of the antagonist (maxillary P2), the occlusal plane of the cheek teeth shows a subtle caudal slant (red dashed line).

Pet rabbit medicine has improved over the last 20 years, especially with regard to better diagnostics and a more targeted therapy for many different health problems. Yet, it still is often restricted as compared to medicine in cats and dogs. One of the main reasons for this limitation is that species-specific differences in anatomy, physiology, and behavior are not sufficiently taken into account. Very frequently encountered diseases in pet rabbits are acquired dental problems that affect all age groups and are equally distributed with respect to sex [1,2,3,4,5,6]. When including radiography as a diagnostic tool, the number of reported patients suffering from malocclusion caused by different pathological periodontal and dental changes reaches up to 88% of the overall clinical population [7,8]. Even among apparently healthy animals, a high prevalence of clinical and radiological abnormalities concerning the teeth has been reported. A survey of 167 pet rabbits that were considered healthy by their owners and that were not receiving any veterinary treatment at the time of the health survey revealed that 40.1% suffered from acquired dental disease [5]. This is due to the fact that rabbits as a prey species in general often show scarcely or too late any outward signs of distress [9]. Hence, affected pet rabbits first appear completely asymptomatic despite the presence of subtle periodontal and/or dental anomalies. Therefore, early symptoms of malocclusion are often overlooked (Figure 1A,B). Often only in cases of severe malocclusions, late symptoms such as anorexia and weight loss get evident to the owner, thus assuming dental problems [4,7,10]. Consequently, first consultation and diagnosis occurs at an advanced stage of the disease, when successful treatment is difficult or even not possible any more [7]. This highlights the importance to routinely integrate effective, noninvasive techniques such as skull radiography, in the clinical examination routine [7,11]. Especially, as valid experience has shown that only about 20% of potential dental pathology can be recognized by means of a thorough intraoral examination [7]. For visualization of the remaining 80% of dental and periodontal pathology a supplementary radiographic evaluation is mandatory [7]. Additionally, objective criteria for a better interpretation of the X-ray images ensures earlier detection of dental diseases and, thus, may help to avoid evitable late-stage scenarios [7,11]. This is the reason why, in 2009, Böhmer and Crossley [11] published species-specific reference lines that can be used on standard skull X-rays. They facilitate a more objective interpretation of malocclusion in the most commonly affected small pet animals [11]. Although a study criticizes the seemingly limited applicability of this practical method by stating that frequent exceptions do exist [12], the use of the occlusal reference line as described by Böhmer and Crossley [11] has proved to be valuable in the majority of cases. In contrast to cephalometric measurements that were found to be not or little indicative for evaluating tooth health in pet rabbits, the anatomical reference lines according to Böhmer and Crossley [11] reliably revealed dental findings even in rabbits that were graded as tooth healthy in previous clinical examinations [13].

In pet rabbits with normal occlusion, one of the anatomical reference lines corresponds to the occlusal plane [7]. Used on the laterolateral skull radiograph, it begins at the most rostral end of the hard palate immediately caudal to the second incisors and extends caudally to pass through the tympanic bulla at approximately one-third of its height (Figure 1C,D). Despite its proven usefulness in most domestic rabbits, the occlusal reference line appears not to be suitable for application in wild rabbits [14]. The quantitative investigation of skull shape in wild and pet rabbits revealed morphological differences that explain this discrepancy [14]. The cranium is generally more quadratic in domestic rabbits, whereas wild rabbits tend to have a longer and flatter skull.

Here, we address the question of why there are some exceptions among domestic rabbits in which the references lines cannot be applied correctly. To do so, we (1) quantify the cranial morphology of a large data set representing the typical variety of pet rabbit patients and (2) identify the morphological features specific for pet rabbits in which the occlusal reference line cannot be applied successfully. This will allow us to test the hypotheses (1) that the occlusal reference line is suitable for a considerable diversity of pet rabbits, but (2) that there are specific cranial phenotypes that necessitate an adapted occlusal reference line.

## 2. Materials and Methods

### 2.1. Data Set and Radiographic Screening

A total of 80 skulls of sexually mature domestic rabbits (*Oryctolagus cuniculus*) were sampled for the present study. Smaller breeds reach sexual maturity around 4–5 months of age, larger breeds at 6 months of age. We chose to focus not on a specific breed, but on the most commonly recorded group called ‘domestic’ in the veterinary clinical records. The breed status in these animals is not known due to frequently loose records or may even be unknown by the owner or veterinary personnel [1]. Our sample represents the typical diversity of pet rabbits in a clinical population, excluding animals with congenital brachygnathia or extremely giant breeds. The animals’ body mass ranged from 0.9 to 6.3 kg. The specimens were radiographed for medical reasons and not for the purpose of this study. Standardized skull radiographs (laterolateral view) of anesthetized specimens were obtained with the mouth closed or open about 1 mm [11]. Obtaining optimal X-ray images is essential for diagnosis and, thus, minimizes the risk of distortion effects on the analysis of cranial morphology.

Using the species-specific anatomical reference lines according to Böhmer and Crossley [11], the individuals were assigned to one of the two following groups: Group Y (N = 58) includes all individuals in which the occlusal reference line could be applied successfully; i.e., the line corresponds to the physiological occlusal plane. Group N (N = 22) includes all individuals in which the occlusal reference line could not be applied correctly; i.e., the line does not correspond to the physiological occlusal plane lying either too high or too low.

First, linear morphometrics was used to evaluate the morphological diversity and to identify distinct features that may explain group differentiation. Next, geometric morphometrics was applied to reveal if global shape differences of the cranium between the groups are associated with the previously identified distinct features.

### 2.2. Linear Morphometrics

**Figure 2:**
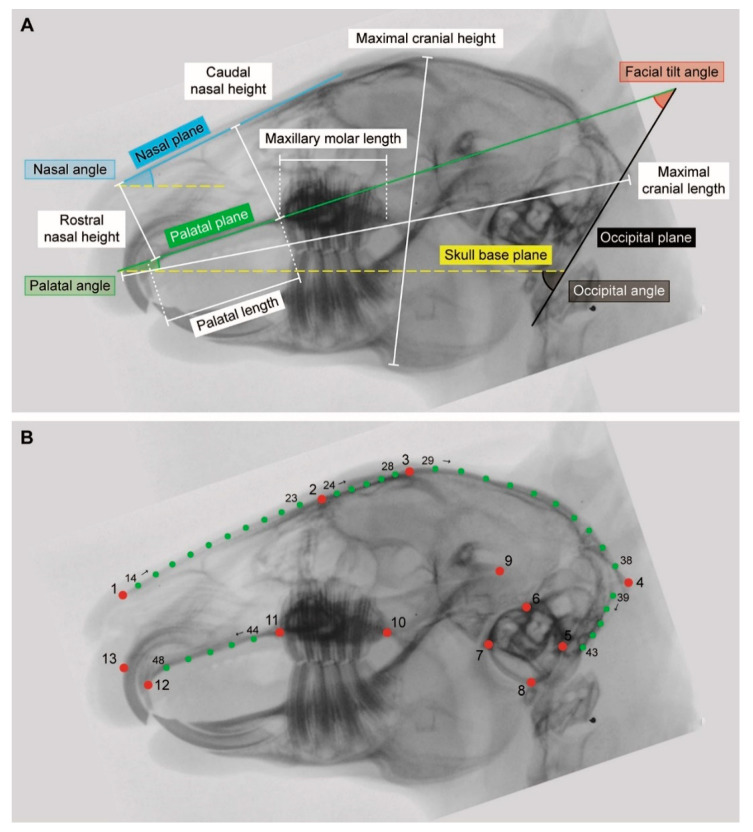
Morphometrics. (**A**) Linear measurements (white) and angles used in the linear morphometric analysis. Planes are indicated with colored lines. Refer also to Table 1. (**B**) Set of 2D landmarks (red) and semilandmarks (green) used in the geometric morphometric analysis. Refer also to Table 2.

Six linear measurements and three angles were obtained to evaluate the diversity in cranial morphology (Figure 2A, Table 1). Maximal cranial length was used to size-normalize the data (see below) and, thus, not further included in the statistical analysis. An additional angle, the occlusal angle, was analyzed separately because it is not independent of the facial tilt and palatal angle. Each measurement was taken three times using the software ImageJ v.1.48 (National Institutes of Health, Bethesda, MD, USA) [15] and the mean value was then calculated.

Prior to analysis, all linear measurements were log_10_-transformed and size-normalized to ensure that variation could be attributed to shape differences, rather than size differences. The log_10_-transformed data (except the measured angles) were regressed against log_10_-transformed maximal cranial length for each specimen. The resulting residuals, i.e., the size-normalized measurements, were used for the subsequent statistical analyses.

### 2.3. Geometric Morphometrics

Thirteen anatomically defined landmarks and 35 sliding semilandmarks (representing five curves) were used to quantify the cranial morphology (Figure 2B, Table 2). The homologous points were digitized onto laterolateral radiological images to obtain the two-dimensional (2D) coordinates using the software tpsDig2 (New York, NY, USA) [17]. The digitalization of the landmarks was performed by a single author (E.B.) in order to prevent interobserver measurement errors. The placement of the landmarks was repeated three times for each individual. The assessment of intraobserver variance revealed that the error is low ensuring reproducibility of the measurements. The collected data were imported in the software R version 3.6.3 (Vienna, Austria) [18] using the ‘geomorph’ package [19]. The obtained landmark and semilandmarks coordinates were superimposed by a generalized Procrustes analysis (GPA) in order to remove nonshape variations caused by position, orientation, and size differences (‘gpagen’ function in R). The procedure standardizes the sample by translating, rotating, and scaling it to a unit centroid size [20,21]. The morphological diversity (MD) is a measure of shape variation among specimens. It was estimated as the Procrustes variance among group Y and group N using the ‘morphol.disparity’ function (‘geomorph’ package). The comparison allowed to assess if the cranial morphology is more or less diverse across both groups.

### 2.4. Statistical Analyses

All subsequent statistical analyses were performed using the software R version 3.6.3 (respective packages are indicated below). Principal components analyses (PCAs) were used to reduce the multidimensionality of the collected variables and to visualize the distribution of the specimens in the morphospace delimited by the PCs. For the linear morphometric data, the PCA was performed using the ‘prcomp’ function in R (‘stats’ package). The ‘factoextra’ package was used to extract and visualize the output of the PCA [22]. For the geometric morphometric data, the PCA was conducted using the ‘plotTangentSpace’ function in R (‘geomorph’ package). The shape differences were visualized with point displacements (‘plotRefToTarget’ function in R). The plots display the landmarks and semilandmarks of the target specimen overlying those of the reference specimen.

Statistical tests were conducted to identify significant morphometric differences explained by the grouping (Y and N, respectively). For the linear morphometric data, multivariate analysis of variance (MANOVA) was used to test if the measured variables differ between the groups (‘manova’ function in R package ‘stats’). The (univariate) ‘t.test’ function in R package ‘stats’ was used to determine if there is a difference in means of skull length to height ratio between the groups. For the geometric morphometric data, Procrustes analysis of variance (ANOVA) was performed to test for significance of shape differences between the groups (‘procD.lm’ function in R package ‘geomorph’).

Nonhierarchical clustering is an unsupervised learning algorithm that tries to find groups and to cluster data based on their similarity. A K-means cluster analysis was performed to reveal the grouping of the studied specimens based on the linear morphometric data (‘kmeans’ function in R package ‘factoextra’). The number of clusters (K) was specified as two (corresponding to the number of groups Y and N, respectively).

The correlation coefficient (Pearson’s R) between the linear measurements was determined to measure the strength of linear association between two variables (‘cor.test’ function in R package ‘stats’).

## 3. Results

### 3.1. Linear Morphometrics

**Figure 3:**
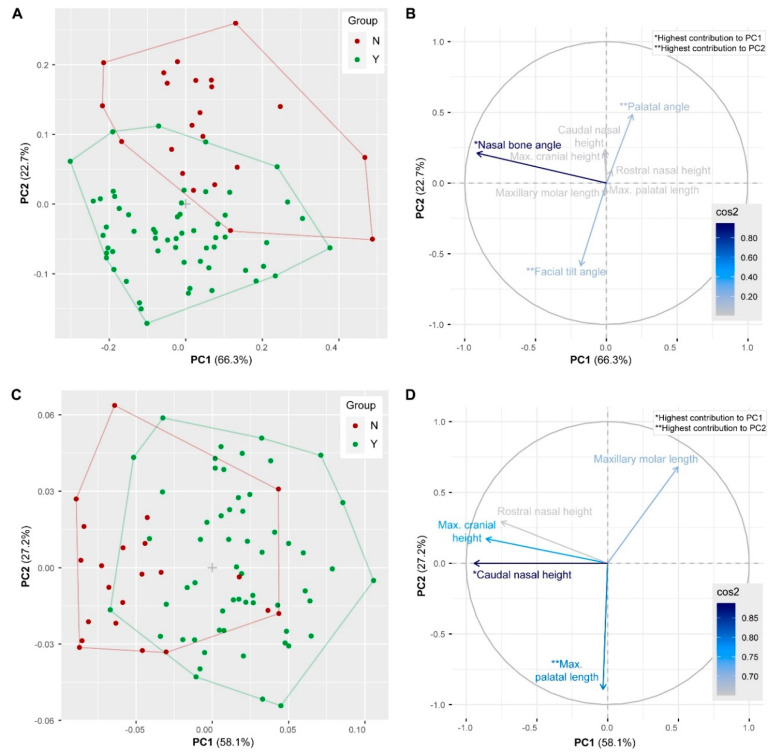
Results of the principal components (PC) analyses. (**A**,**B**) Scatterplot and contributions of the variables considering all measurements. (**C**,**D**) Scatterplot and contributions of the variables considering linear measurements only. The percentage of explained variances by each PC is indicated in parentheses. Arrows represent the squared loadings of the variables and the color intensity is proportional to the value of the loading. Variables that are closer to the correlation circle contribute more to the principal components.

The results of the linear morphometric analysis reveal that specimens of group Y and group N differ in certain, but not all cranial dimensions. Considering all variables (angles and size-corrected linear measurements), the first and second PC account for 89.0% of the total variance in the sample. The morphospace delimited by PC1 and PC2 displays two slightly overlapping clusters representing group Y and group N (Figure 3A). The separation is mainly along PC2 with specimens of group Y having negative PC2 scores and specimens of group N having positive PC2 scores. PC1 distinguishes specimens according to the nasal angle (Figure 3B). PC2 separates specimens according to the palatal angle and facial tilt angle. High PC2 scores are associated with a large palatal angle, whereas low PC2 scores are characterized by large facial tilt angles. MANOVA detected significant differences between group Y and group N for the palatal angle, the caudal nasal height, the facial tilt angle and the maximal cranial height (*p* < 0.0001) and for the maxillary molar length and rostral nasal length (*p* = 0.01) (Table 3).

Considering all size-corrected linear measurements (excluding the angles), the first and second PC account for 85.3% of the total variance in the sample. The morphospace delimited by PC1 and PC2 shows two largely overlapping clusters representing group Y and group N (Figure 3C). There is a slight separation along PC1 with specimens of group Y tending to have positive PC1 scores and specimens of group N tending to have negative PC1 scores. PC1 distinguishes specimens mostly according to caudal nasal height (Figure 3D). High PC1 scores are associated with a low caudal nasal height, whereas low PC1 scores are characterized by a high caudal nasal height. PC2 separates specimens according to maximal palatal length.

### 3.2. Skull Length and Height

The linear morphometric analysis indicates that the skull height also plays a role in the differentiation of the two groups. Maximal cranial length in relation to maximal cranial height shows significant differences between both groups (t-test: t = −5.70, df = 31.64, *p* < 0.0001). Specimens of group N tend to have shorter skulls in relation to their skull height (Table 4).

### 3.3. Nasal Height

The linear morphometric analysis revealed that the height of the nasal region is involved in group differentiation. In contrast to the caudal nasal region, the rostral nasal region is a weak descriptor for group identification (Table 5). The k-means cluster analysis using the rostral nasal region assigned only 22.7% of group N specimens to one cluster and 51.7% of group Y specimens to another cluster. Using the caudal nasal region, the k-means cluster analysis revealed that the variable assigns each about 80% of specimens to the correct group (Table 5).

### 3.4. Palatal and Facial Tilt Angle

As indicated by the linear morphometric analysis, the palatal angle and the facial tilt angle are the good descriptors for group identification. The k-means cluster analysis using the palatal angle assigned 100% of group N specimens to one cluster and 87.9% of group Y specimens to another cluster (Table 5). By contrast, the k-means cluster analysis using the facial tilt angle assigned only 77.3% of group N specimens to one cluster and 62.1% of group Y specimens to another cluster.

**Figure 4:**
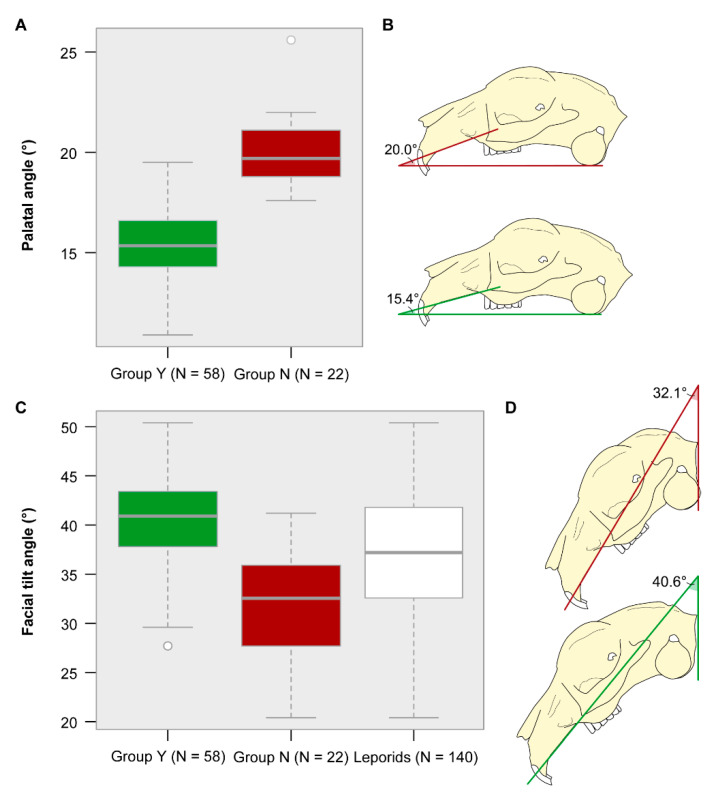
Variance in palatal angle and facial tilt angle. (**A,C**) Boxplot and (**B,D**) schematic visualization of the mean angle for each group. Both, the palatal angle and the facial tilt angle, are significantly different between group Y and group N. (**C**) The variance in facial tilt angle among leporids (data from Kraatz et al. [23]) lies within the combined variance observed in pet rabbits (this study).

The palatal angle is significantly different between both groups (Table 3). The mean angle of group Y is 15.4° and the mean angle of group N = 20.0° (Figure 4A,B). Specimens of group Y have a rather flat palate, whereas the palate in specimens of group N is steep.

There is a significant difference in the facial tilt angle between both groups (Table 3). The mean angle of group Y is 40.6° and the mean angle of group N is 32.1° (Figure 4C,D). The cranium in specimens of group Y is less steeply inclined as compared to that in specimens of group N. The variance in facial tilt angle among leporids (data from Kraatz et al. [23]) equals the combined variance observed in domestic rabbits (this study) (Figure 4C).

**Figure 5:**
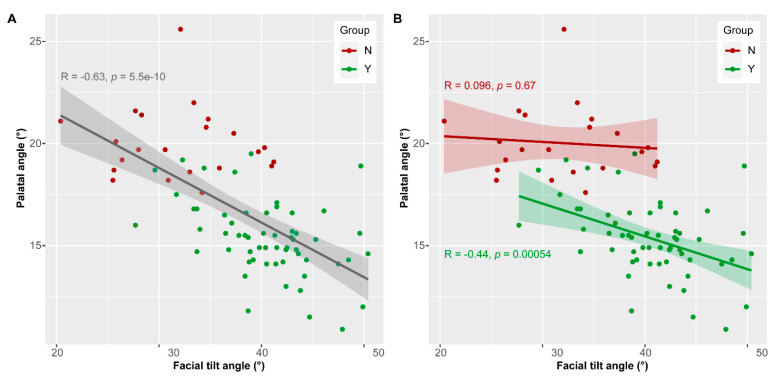
Relation between facial tilt angle and palatal angle. (**A**) Linear regression considering all data. (**B**) Linear regression considering the two groups separately.

Although both, palatal angle and facial tilt angle, contribute to group separation (Figure 3A,B), the k-means cluster analysis (see above) revealed that the latter is a weaker descriptor for group identification. This suggests that the relation between the two angles may not be linear in group Y and group N. Considering all data, the Pearson’s correlation test revealed a negative linear relationship between facial tilt angle and palatal angle (R = 0.63, *p* < 0.0001) (Figure 5A). This means that large facial tilt angles are associated with small palatal angles. Considering the two groups separately, there is no linear relation between facial tilt angle and palatal angle in group N (R = 0.096, *p* = 0.68) and a negative linear relationship in group Y (R = −0.44, *p* = 0.0005) (Figure 5B). The specimens of group N are outstanding because they display varying facial tilt angles, but rather large palatal angles.

### 3.5. Occipital Angle

**Figure 6:**
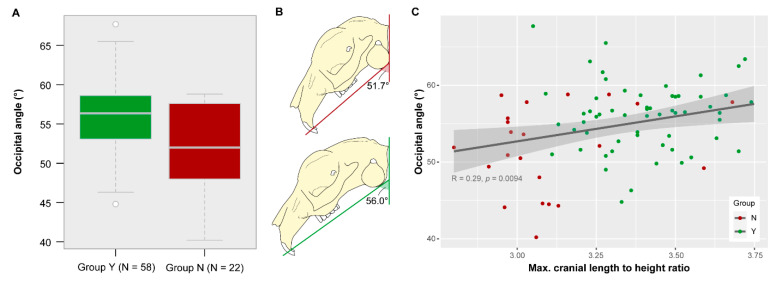
Variance in occipital angle. (**A**) Boxplot and (**B**) schematic visualization of the mean angle for each group. It is significantly different between group Y and group N (*p* = 0.003). (**C**) Relation between occipital angle and maximal cranial length to height ratio.

There is a significant difference in the angle between skull base and occiput between both groups (*t*-test: t = 3.1905, df = 31.286, *p* = 0.003). The mean angle of group Y is 56.0° and the mean angle of group N is 51.7° (Figure 6A). Taking the occiput in vertical position as a reference line, the skull base in specimens of group Y is less steeply inclined as compared to that in specimens of group N (Figure 6B). Yet, the variable is not a good descriptor for group identification. The k-means cluster analysis using the occipital angle assigned 67.5% to the correct group (Y or N, respectively). Of group Y, 30% were not correctly identified. Of group N, 40% were misidentified.

Considering all data, the Pearson’s correlation test detected a significant, but weak negative linear relationship between the occipital angle and the palatal angle (R = −0.29, *p* = 0.008). Considering the two groups separately, there is neither a linear relation between the occipital angle and the palatal angle in group N (R = 022, *p* = 0.32) nor in group Y (R = −0.12, *p* = 0.36).

There is a significant, but weak positive linear relationship between the occipital angle and the maximal cranial length in relation to height (R = 0.29, *p* = 0.009) when considering all data (Figure 6C). Specimens of group N tend to have shorter skulls in relation to their skull height and this cranial morphology tends to be associated with rather lower angles. The skull base in specimens of group N is steeply inclined (Figure 6B).

### 3.6. Geometric Morphometrics

**Figure 7:**
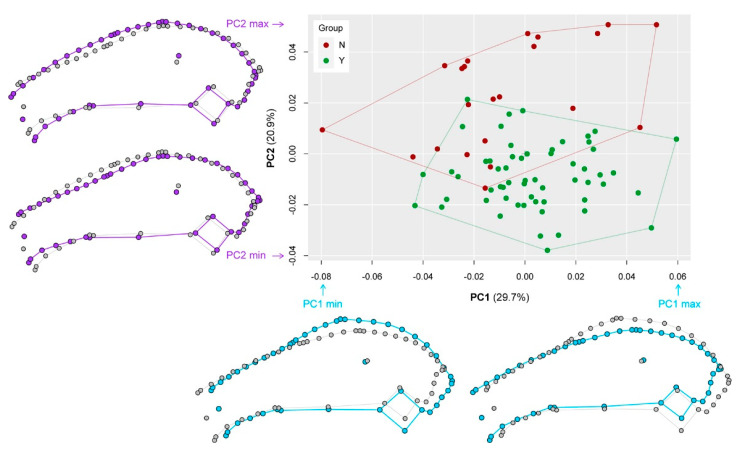
Results of the geometric morphometric analysis. The scatterplot displays the distribution of the sampled specimens in the morphospace represented by the first two principal components (PC1 and PC2). The percentage of explained variance by each PC is indicated in parentheses. The outlines visualize the shape differences between the extreme configurations (min = minimum, max = maximum) along PC1 and PC2.

The geometric morphometric analysis explains global shape differences between the groups and the results support the previous observations based on linear morphometric data on cranial differences between groups Y and N. The specimens of group Y and group N differ in overall cranial morphology (Figure 7). The first and second PC account for 50.6% of the total variance in the sample. The morphospace delimited by PC1 and PC2 displays two slightly overlapping clusters representing groups Y and N (Figure 7). The separation is mainly along PC2 with specimens of group Y having negative PC2 scores and specimens of group N having positive PC2 scores. PC1 distinguishes specimens according to the shape of the ocular region and the braincase (light blue outlines in Figure 7). High PC1 scores are associated with a long nasal region, a flat ocular region, and a short braincase, whereas low PC1 scores are characterized by a short nasal region, a high ocular region, and a long braincase. PC2 separates specimens according to the lateral orientation of the palate and the shape of the caudal nasal region (purple outlines in Figure 7). High PC2 scores are associated with a high nasal region and a steeply inclined palate. Low PC2 scores are characterized by a low nasal region and a plane palate. Procrustes ANOVA detected significant differences (F = 4.0585, *p* = 0.01) between group Y and group N.

Taking the mean of the entire sample, the comparison of the Procrustes variance revealed that the skull disparity is larger in group N (MD_N_ = 0.0029) than in group Y (MD_Y_ = 0.0017). Specimens of group N cover a slightly larger area of the morphospace in contrast to specimens of group Y (Figure 7). The difference is statistically significant (*p* = 0.002).

## 4. Discussion

The masticatory apparatus of rabbits with its ever-growing teeth is a functional system that is highly sensitive to alterations because of the vital balance between dental growth and wear [3,7,24]. Tooth overgrowth, for example, leads to a change in the natural position of dental contact—a situation referred to as malocclusion. In order to assess malocclusion in pet rabbits, it is necessary to define the species-specific normal or physiological occlusion (normocclusion) in which the dentition functions properly. The anatomical reference lines according to Böhmer and Crossley [11] provide an objective criterion to visualize the physiological occlusal plane of the molars in lateral view (Figure 1C,D). Any deviation from this plane indicates abnormal clinical crown height of the molars and the incisors. The present study confirms that the applicability of the occlusal reference line is not restricted to one specific skull morphology in rabbits as we detected considerable skull shape diversity within group Y; i.e., morphospace distribution (Figure 3A and Figure 7). Nevertheless, there are some exceptions and the present results show how to identify them.

### 4.1. Skull Shape Diversity in Pet Rabbits

The quantification of the cranial morphology revealed differences among the specimens of our sample of pet rabbits. These differences in skull shape largely characterize the two different groups, that is group Y in which the occlusal reference line could be applied successfully and group N in which the occlusal reference line could not be applied correctly. As compared to group Y, individuals of group N tend to have a short skull in relation to their skull height, the caudal part of the nasal is rather high and a steeply inclined cranium that is quantified by a small facial tilt angle, a small occipital angle, and a large palatal angle, in particular. Our study revealed that the palatal angle is the best descriptor for group identification (100% of group N specimens). This suggests that pet rabbits with a rather large palatal angle (>18.8°) are not suitable for the successful application of the occlusal reference line as described by Böhmer and Crossley [11]. In specimens with a large palatal angle, the maxillary cheek tooth row lies beyond the physiological occlusal plane as defined by the anatomical reference lines. This can lead to a misinterpretation of the dentition.

The diversity in skull shape is slightly higher in group N as compared to group Y suggesting that there are some rather extreme cranial morphologies (Figure 7). Nevertheless, there is also some overlap between both groups indicating that the overall skull dimensions of group N as quantified by the linear measurements is not extremely different from group Y (Figure 6C). For instance, there is no significant difference in maximal palatal length in our sample of pet rabbits (Table 3). As explained above the main difference arises from the dorsal curvature of the cranium as measured by the facial tilt angle, the caudal nasal height, and the palatal angle (Figure 6A; Table 3).

### 4.2. Evolutionary Transformations of the Rabbit Skull

**Figure 8:**
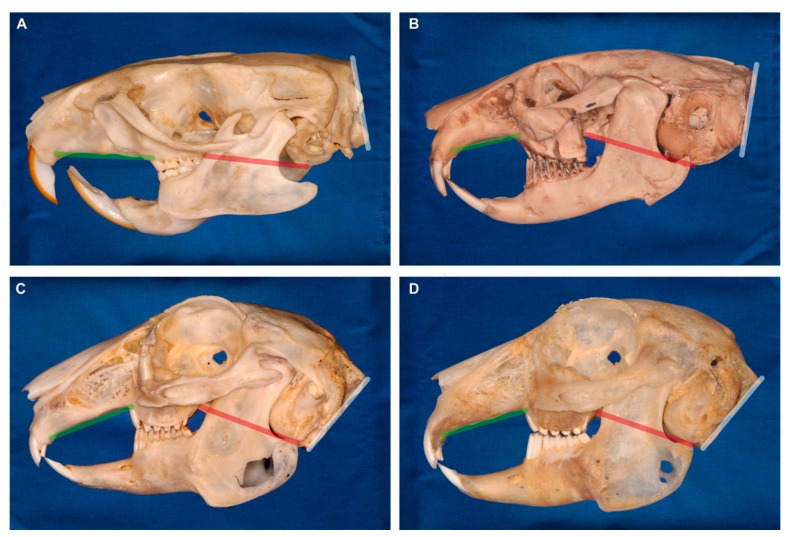
Cranial flexion in rodents and the sister taxon lagomorphs (ochotonids and leporids). The red line represents the orientation of the basicranium. The green line indicates the palatal plane. The white line shows the orientation of the occiput and the foramen magnum. (**A**) Rat (rodent). (**B**) Pika (ochotonid). (**C**) Wild rabbit (leporid). (**D**) Pet rabbit (leporid). Note the pronounced flexion in lagomorphs. The skulls are scaled to same length and oriented with the cranial base plane being horizontal.

The leporid skull exhibits a number of morphological synapomorphies such as the extensive fenestration of the maxillary bone and a pronounced flexion of the head [25,26,27] (Figure 8). Yet, the functional significance of these modifications remains largely unclear to date. It is still under debate if the cranial features are linked to the development of the characteristic saltatorial mode of locomotion in rabbits or if they are associated with mastication. For example, on the one hand, the fenestrated maxilla may be interpreted as locomotor adaptation because it reduces the mass of the skull which facilitates stabilization of the head under dynamic loading [25]. On the other hand, it has been shown that the fenestration reflects the normal lack of transmission of masticatory forces [26,28]. Artificial loading of the vertical plane of the maxilla of the rabbit induces the formation of compact bone in the area that is normally fenestrated [26].

In contrast to the straight skull in rodents (e.g., rats) and the moderately curved skulls in ochotonids (pikas; the sister group of rabbits and hares), an increasingly more pronounced flexion of the head in lateral view has evolved in lagomorphs [27,29,30,31,32] (Figure 8). The rostral region is rotated ventrally relative to the basicranium that is caudally likewise tilted with the occipital region (including the occipital foramen) pointing ventrally (Figure 8C,D). This evolutionary change is thought to be linked to the characteristic saltatorial mode of locomotion in rabbits because it adjusts the orbital orientation and the orientation of the occipital foramen to the vertically hold cervical vertebral column [23,27,31,32]. Using the facial tilt angle, it has been shown that among leporids (rabbits and hares), generalist taxa have less tilted crania (i.e., larger angles; mean facial tilt angle = 44.0°) than saltatorial ones (smaller angles; mean facial tilt angle = 37.2°) [23,27]. There is morphogenetic evidence showing that artificial manipulation of cranial growth in rats results in a rabbit-like moderately curved head [33]. In rabbits, experimentally induced premature closure of cranial sutures leads to an abnormally increased curvature of the skull [34,35].

The study by Moss [36] adding a functional aspect to these observations, revealed that the artificial removal of limb pairs is capable of altering the skull morphology in rats. In “bipedal” animals that lack forelimbs, the rostral region rotated downwards during development [36]. Cranial flexion and orbit orientation appear also to play a role in primate evolution towards bipedal locomotion [37,38]. However, basicranial flexion alone would be sufficient to compensate for a more upright or bipedal body posture. Consequently, the ventral rotation of the rostral region in rabbits could be linked to other functional differences instead.

In our pet rabbit sample, the specimens of group Y (mean facial tilt tangle = 40.6°) lie within the variance of the facial tilt angle among leporids (Figure 4C). In contrast, half of the specimens of group N have lower facial tilt angles than measured in leporids (Figure 4C). Consequently, the skulls in some domestic rabbits are more steeply inclined when taking the occiput in vertical position as a reference line (Figure 4D). However, the locomotor mode does not differ in our pet rabbit sample. Another line of evidence suggests that diet and, thus, masticatory forces influence the morphology of the premaxilla and the diastema in herbivorous mammals in general [39]. In lagomorphs, the evolution of gnawing incisors is closely associated with transformations of the rostral cranium and the skull arrangement in regard to the incisors [40,41]. Rabbits lack canines and have a long diastema between the incisors and the cheek teeth [32]. It separates two masticatory functions, biting in the rostral part of the oral cavity and grinding in the caudal part [32,42,43]. The tips of the sharply curved incisors in rabbits are directed caudally (opisthodont), which is advantageous for cutting functions [32]. Potentially, the occlusal forces applied in incisal biting may only be possible in combination with the ventral rotation of the rostral region.

A pronounced inclination of the skull may be due to either a general ventral positioning of the head (small occipital angle) or a ventral inclination of just the rostral region of the skull (large palatal angle). Since the occipital angle is not as different as the palatal angle in our sample, it appears that the more pronounced curvature of the skull in group N is mainly due to a transformation of the rostral region. The large palatal angles in group N result in skulls that are more strongly curved in ventral direction (Figure 4B). Our landmark analysis visualizes that the caudal skull shape between group N and group Y is less different as compared to the rostral region (comparison of the outlines PC2 min vs. PC2 max, in particular, in Figure 7). This suggests that domestication may have led to an overemphasis of the dorsal curvature of the skull in some pet rabbits. This affects the position of the incisors relative to the skull and, consequently, influences the applicability of the reference lines (Figure 1C,D).

### 4.3. Skull Development and Palate

In contrast to the caudal region, the rostral region of the skull in mammals appears to bear greater evolutionary flexibility [44]. A major factor contributing to skull shape diversity involves the angulation of the rostrum relative to the cranial base [45]. The palatal angle (also referred to as prebasial angle and basicranial-basifacial angle, respectively, in other works) is a measurable proxy for the degree of this angulation. According to Hofer [46], three types can be distinguished: (1) orthorhynchy: the rostral region is in line with the basicranium; (2) airorhynchy: the rostral region is deflected in dorsal direction relative to the basicranium; (3) clinorhynchy: the rostral region is deflected in ventral direction relative to the basicranium. Among domestic mammals, dogs show an enormous craniofacial diversity with extreme airorhynchy or bulldog-type skull conformation and strong clinorhynchy or bull terrier-type skull conformation [47,48,49]. Rabbits, in general, are characterized by clinorhynchy. Yet, the present study revealed that the palatal angle is variable among pet rabbits. The detected ventral inclination of the rostral region in the skull of specimens from group N may be due to artificial selection. However, the nasal angle is not significantly different between both group N and group Y, which suggests that the angulation of the rostrum relative to the cranial base is mainly restricted to the palatal bone.

The hard palate is known to vary morphologically in response to variation in masticatory stresses [50,51,52]. The different tongue pressures on the palate and the duration of tongue–palate contact are thought to influence the development of the bone [51]. In comparison to animals fed on a hard diet, the hard palate plane is more distant from the cranial base plane in animals fed a soft diet, resulting in a descended orientation of the palate [51,53]. The epigenetic action of muscles, such as the tongue and the muscles of the masticatory apparatus, have the potential to influence skull development [50,54]. Holtgrave and Müller [55] showed that the surgical repositioning of the superficial masseter muscle in young rabbits resulted in a permanent alteration of the mandible. The mandibles in the manipulated individuals were significantly shorter and had an enlarged gonion angle (angle between the base of the mandible and the caudal border of the ramus) [55]. Consequently, it may be possible that the skull shape in group N (the large palatal angle, in particular) is the result of muscle-induced phenotypic plasticity due to different diet. A future study that investigates the relation between skull morphology, diet, and prevalence of malocclusion will shed light on the role of the palatal bone in pet rabbits.

### 4.4. Outlook: Breeding for Good Dental and Oral Health

In view of the present results, it would be interesting to reveal if the palatal angle is related to acquired dental disease in pet rabbits. If such a correlation between cranial morphology and oral health exists, it necessitates to consider this parameter when selecting animals for breeding with the aim that they do not develop dental disease. The palatal angle may also be the result of diet that has been fed during early postnatal ontogeny. In the case of a relation to acquired dental disease, this would suggest to improve feeding of young animals.

## 5. Conclusions

The present study showed that there is considerable diversity in skull shape among the typical pet rabbit (of no specific breed). The domestication of rabbits—most likely with a single origin from wild populations of France [56,57,58]—has led to a very high phenotypic diversity with more than 200 breeds recognized worldwide [57]. The morphological variation is reflected in a wide variety of commercial and laboratory uses. Nevertheless, the applicability of the occlusal reference line is not significantly influenced by variance in parameters such as the length of the maxillary molar row or of the palatal bone. It is slightly affected by the caudal height of the nasal bone and the cranial length to height ratio. In pet rabbits with a rather high caudal nose and a short skull in relation to their skull height, the occlusal reference line is not always applicable. However, these parameters explain only partially why in some cases the occlusal reference line cannot be applied. The most substantial parameter that allows to predict if the occlusal reference line is applicable or not is the palatal angle. If the angle between the skull base and the palatal bone is larger than 18.8°, the occlusal plane of the molars is shifted outside the occlusal reference line. In these cases, they are not suitable for successful objective interpretation of norm- and malocclusion.

## Figures and Tables

**Table 1:** Definition of the established measurements and planes used in the linear morphometric analysis. The linear measurements and the angles were obtained on laterolateral X-ray images of the skull. Refer also to Figure 2A.

Linear Measurement	Definition
Caudal nasal height	Distance (perpendicular to the nasal plane) between the caudal nasal roof and the palate (at the rostral intersection between maxillary bone and first maxillary cheek tooth P2)
Maximal cranial length	Maximal distance between the most rostral end of the upper incisor and the occipital protuberance (crossing the most rostral point of the hard palate)
Maximal cranial height	Maximal distance between the mandibular facial incisure to the most dorsal point of the frontal bone
Maxillary molar length	Maximal distance between the first maxillary cheek tooth P2 and the last maxillary molar M3
Palatal length	Distance between the most rostral end of the palate (at the intersection between second maxillary incisor I2 (peg tooth) and maxillary bone) and the rostral intersection between maxillary bone and first maxillary cheek tooth P2
Rostral nasal height	Distance (perpendicular to the nasal plane) between the rostral nasal roof (at the most rostral end of the nasal bone) and the palate
**Plane**	
Nasal plane	Tangent to the nasal bone
Occipital plane	Tangent to the occiput (great foramen)
Palatal plane	Tangent to the hard palate (without its most rostral part which slopes downwards)
Skull base plane	Line connecting the rostral end of the hard palate immediately caudal to the second incisor and the ventral border of the tympanic bullae
**Angle**	
Facial tilt angle	Angle between the palatal plane and the occipital plane
Nasal angle	Angle between the nasal plane and the skull base plane
Occipital angle	Angle between the occipital plane and the skull base plane
Palatal angle	Angle between the palatal plane and the skull base plane

**Table 2:** Definition of the landmarks (LM) and semilandmarks (semiLM) used in the geometric morphometric analysis. The homologous points were applied to laterolateral X-ray images of the skull. Type I, II, and III landmarks sensu Bookstein [16]. Refer also to Figure 2B.

#	Type	Definition
**LM**		
1	II	Most rostral point of nasal bone
2	I	Intersection between nasal bone and rostral orbital roof
3	I	Intersection between frontal bone and caudal orbital roof
4	II	Most caudal point of occipital protuberance
5	II	Most caudal point of tympanic bulla
6	II	Most dorsal point of tympanic bulla
7	II	Most rostral point of tympanic bulla
8	II	Most ventral point of tympanic bulla
9	III	Center of temporomandibular joint
10	I	Caudal intersection between maxillary bone and last maxillary molar M3
11	I	Rostral intersection between maxillary bone and first maxillary cheek tooth P2
12	I	Intersection between second maxillary incisor I2 (peg tooth) and premaxillary bone
13	I	Rostral intersection between first maxillary incisor I1 and premaxillary bone
**SemiLM**		
14–23	-	Nasal bone (curve between LM1 and LM2)
24–28	-	Orbital roof (curve between LM2 and LM3)
29–38	-	Dorsal braincase (curve between LM3 and LM4)
39–43	-	Occiput (curve between LM4 and LM5)
44–48	-	Palate (curve between LM11 and LM12)

**Table 3:** MANOVA results of the linear morphometric data.

Group Y vs. Group N	F Value	*p*-Value
Palatal angle	90.654	<0.0001 *
Caudal nasal height	46.971	<0.0001 *
Facial tilt angle	43.599	<0.0001 *
Maximal cranial height	42.792	<0.0001 *
Maxillary molar length	6.6609	0.01 *
Rostral nasal height	6.1633	0.01 *
Nasal bone angle	0.6396	0.4263
Maximal palatal length	0.2096	0.6483

Asterisk (*) indicates statistical significance.

**Table 4:** Maximal cranial length (l) to height (h) ratio.

l/h Ratio	Group Y (N = 58)	Group N (N = 22)
Minimum	3.05	2.8
Mean	3.40	3.11
Maximum	3.74	3.68
Variance	0.03	0.05

**Table 5:** Results of the k-means cluster analyses testing the variables that contribute most to the group differentiation in the linear morphometric morphospace.

Variable	Correctly Assigned to Group N	Correctly Assigned to Group Y
Rostral nasal height	22.7%	51.7%
Caudal nasal height	81.8%	82.8%
Facial tilt angle	77.3%	62.1%
Palatal angle	100%	87.9%
